# Transcapular Nerve Block and Subacromial Bursa Injection in Shoulder Pain in Fibromyalgia in a Female Patient: A Case Report

**DOI:** 10.7759/cureus.79755

**Published:** 2025-02-27

**Authors:** Maimuna F Ahmed, Raveen K Aujla, Grigory Karmy

**Affiliations:** 1 General Medicine, Dr. Nandamuri Taraka Rama Rao (NTR) University of Health Sciences, Hyderabad, IND; 2 Interdisciplinary Medical Sciences (IMS), Western University, London, CAN; 3 Family Medicine, Faculty of Health Sciences, McMaster University, Hamilton, CAN

**Keywords:** bursitis, central sensitization, fibromyalgia, fibromyalgia syndrome, fms, high-frequency nerve blocks, shoulder pain, subacromial bursitis, subacromial injection, transcapular nerve block

## Abstract

This case report investigates the use of transcapular nerve blocks and subacromial bursa injections with a local anesthetic to manage shoulder pain in a 53-year-old female patient with fibromyalgia. The patient, who was diagnosed with fibromyalgia, bilateral rotator cuff tendinosis, and subacromial bursitis after a fall, showed improvement in pain and function after 8 weeks of treatment with the combined injections. Her visual analogue scale (VAS) score was reduced from 9 to 2, and her range of motions returned to normal. This significant improvement supports the hypothesis that combining the administration of a local anesthetic with transcapular nerve blocks and subacromial bursa injections may effectively reduce shoulder pain in fibromyalgia patients. Future large-scale observational and randomized controlled trials are needed to validate these results.

## Introduction

Fibromyalgia (FM) is associated with various conditions, including bursitis, chondromalacia, temporomandibular joint dysfunction, pelvic pain, vertigo, mitral valve prolapse, spastic colon, and other symptoms like sensory symptoms, weakness, sinus issues, swollen glands, tinnitus, thyroid problem, tachycardia, chronic cough, constipation, and diarrhea [1]. Central sensitization, a key mechanism in FM, amplifies pain perception by increasing the excitability in the central nervous system. This increased sensitivity makes individuals more prone to experiencing pain from concomitant pathologies like bursitis, which can further exacerbate chronic shoulder pain. Therefore, effective management of bursitis in FM patients is crucial to improving overall pain and function. 

Bursitis is the term used to describe the inflammation of the bursa, a fluid-filled sac that reduces friction between tissues such as tendons and bones. The subacromial bursa, located beneath the acromion and above the rotator cuff tendons, is crucial in facilitating shoulder movement. However, it is susceptible to inflammation due to repetitive use, acute trauma, impingement, calcific deposits, infection, autoimmune diseases, and even vaccination. In fibromyalgia patients, where shoulder pain is frequent, it is essential to recognize and treat fibromyalgia-associated bursitis. Bursitis was treated with needling, puncturing, heat, X-ray therapy, ultrasound waves, local anesthetic injections, and cortisone injections. Platelet-rich plasma (PRP) for treating pain associated with bursitis requires more research. In contrast, the administration of hyaluronic acid has yet to prove its efficacy, and subacromial decompression has shown no improved outcomes. The current standard treatment for bursitis is physiotherapy, anti-inflammatory drugs, and partial bursectomy [2]. However, when patients with functional somatic syndromes (FSS) like FM undergo shoulder surgery postoperatively, they tend to have a less-favorable passive range of motion, higher opioid consumption, and increased healthcare costs compared to patients without FSS, though there is an improvement from baseline [3].

The subacromial bursa's role as a cause or consequence of tendinitis is controversial, but its healing properties are attributed to vasculature, growth factors, and various stem cells. In vitro studies have demonstrated that the subacromial bursa helps heal rotator cuff tears [2]. A local anesthetic injection that reduces the inflammation in the bursa can stimulate the bursa to return to its healing function.

To provide an alternative, minimally invasive treatment, this case report examines the administration of a transcapular nerve block and a subacromial injection with a local anesthetic to treat shoulder pain caused by fibromyalgia-associated bursitis. A transcapular nerve block involves injecting a local anesthetic into the suprascapular nerve, which supplies sensory and motor innervation to the shoulder. This block reduces pain transmission, improving mobility and function. A subacromial bursa injection, on the other hand, directly targets inflammation within the bursa, potentially restoring its healing properties. While local anesthetic injections have been used to manage bursitis, their specific role in FM-associated bursitis remains underexplored. This report will provide valuable insight into the effectiveness of the novel approach by analyzing the mechanism of action of local anesthetics in bursitis when combined with transcapular nerve blocks.

## Case presentation

An unemployed 53-year-old Canadian patient presented at a Clinic in Ontario with the chief complaint of pain all over the body. The history of presenting illness was widespread pain in the neck, shoulders, lower back, hips, and knees, numbness in both her hands and feet, headaches, flashing light in the eyes, tinnitus, abdominal pain, urinary stress incontinence, sleep disturbances, and depression. Past medical history included a history of slipping and falling on ice in 2017, injuring her abdomen muscles on the left side, and developing bruises in the same area. On physical examination, bilateral rotator cuff bursitis was diagnosed on examination of both shoulders, with restriction of abduction and tenderness over multiple structures, mainly the supraspinatus, subscapularis, and subacromial bursa. The capsular syndrome was ruled out by no medial and lateral rotation restriction and no history of pain in the deltoid region. No restriction of passive movements ruled out rotator cuff tendinopathy. The patient had a pain score of 57/70 on the brief pain inventory, which delineates her pain. The self-reported leeds assessment of neuropathic symptoms and signs (S-LANNS) pain score questionnaire helps to identify the neuropathic element in an individual's pain, scoring above 12, indicating a neuropathic element was present [4]. On the Hospital Anxiety and Depression Scale (HAADS), she scored 12 for depression, indicating a definite case, and 8 for anxiety, indicating a moderate case. The patient's opioid risk tool score was 6, indicating a moderate risk with a predicted rate of opioid misuse of 28%.

Along with the nerve block and subacromial injections, she was prescribed Gabapentin, five tablets of 300 mg at bedtime, and two extra strength Advil at bedtime as required, which was started before the nerve block injections. She tried acupuncture and chiropractic treatments in the past, which eased the pain for some time. However, the relief was temporary, and the pain returned after a few days. Her past medical history includes bilateral carpal tunnel syndrome and left bundle branch block.

After obtaining informed consent,12.5 mg of a transcapular nerve block and 7.5 mg of a subacromial bursa injection were performed with 0.5% Xylocaine injection on each side, respectively. The patient responded significantly well to the injection. The patient reported a score of two out of 10 after the injections on the VAS, indicating a substantial reduction in pain. The patient was advised to return for weekly injections. At eight weeks follow-up, she reported significant pain improvement with less stiffness and better function. The ultrasound and X-ray of the shoulder came back normal, indicating no structural damage. Her widespread pain was reduced by 60%, which significantly improved her quality of life. The patient tolerated the injections well. No adverse events were observed.

## Discussion

**Figure 1:**
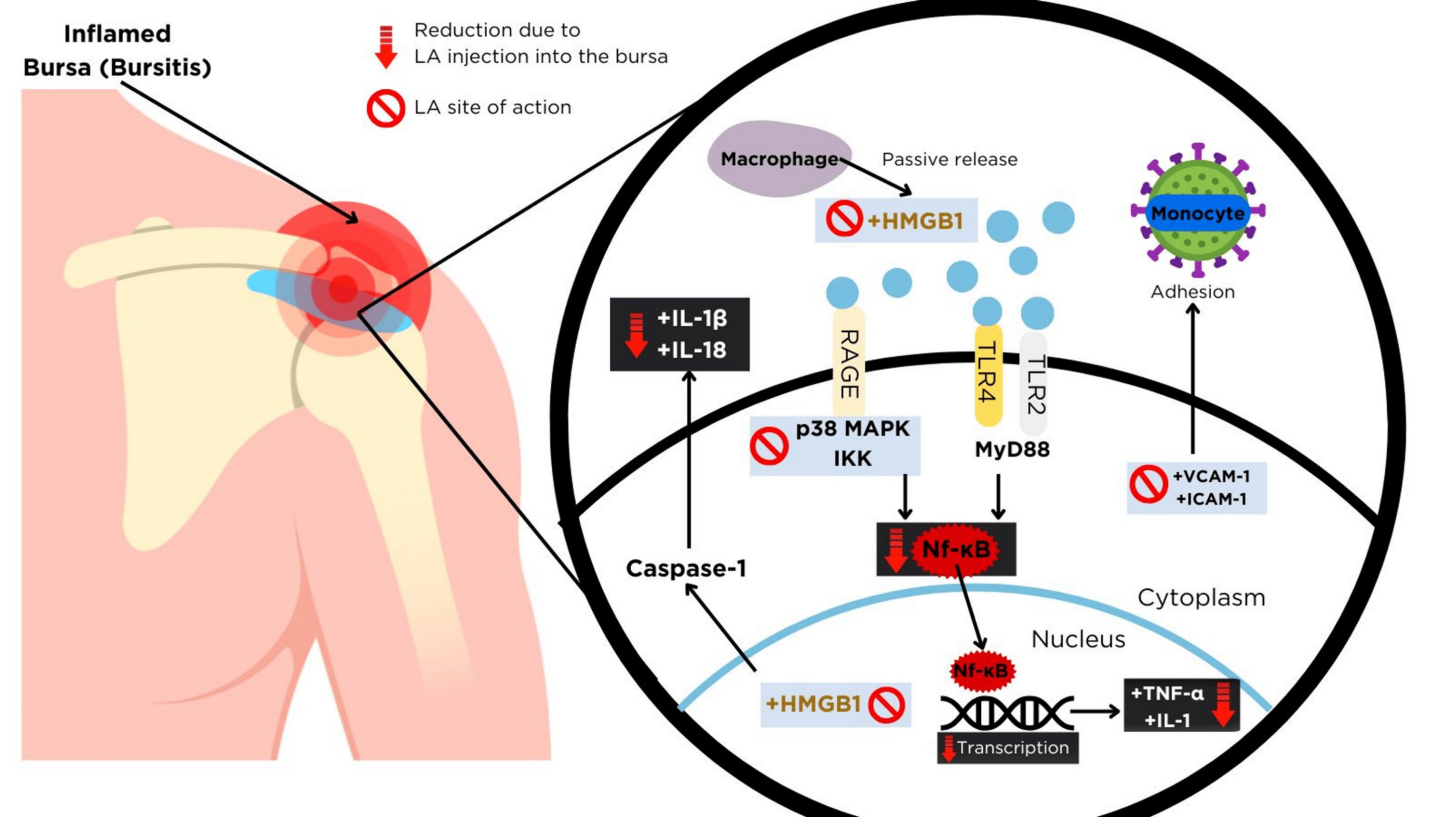
Mechanism of action of subacromial bursa injection with local anesthetic in shoulder pain in fibromyalgia. HMGB1: High Mobility Group Box Protein 1; RAGE: Receptor for Advanced Glycation End Products; TLR: Toll-Like Receptor; DAMPs: Damage-Associated Molecular Patterns; p38 MAPK: Mitogen-Activated Protein Kinase; NF-κB: Nuclear Factor kappa-light-chain-enhancer of activated B cells; VCAM-1: Vascular Cell Adhesion Molecule-1; ICAM-1: Intercellular Cell Adhesion Molecule-1; TNF-α: Tumor Necrosis Factor-alpha; IL-8: Interleukin-8; IL-1: Interleukin-1; IL-1β: Interleukin-1β; IL-18: Interleukin-18. The image was created using canva.com.

Figure 1 explains the mechanism of action of intrabursal injection with local anesthetic in shoulder pain in fibromyalgia patients. Local anesthetic injection into the bursa can reduce the inflammation of bursitis by the following mechanism.

The available literature about transcapular injection and subacromial bursa injection combination is scarce, however, these injections are studied separately in shoulder pain. A retrospective survey-based study showed local anesthetic injection with 1 % procaine into the subacromial bursa followed by physical exercises or without, showed good response in 43 %, fair in 19%, and poor response in 38%of chronic stage of subacromial bursitis. The good response was no pain, recurrence, or limitation of shoulder movements. Fair was some limitation of one or more movements but no recurrence of severe pain or spasms [5]. Another study indicated that subacromial injection with a local anesthetic and steroids showed efficacy in treating bursitis [6]. Potential complications from intrabursal steroid injection include allergic drug reactions, subcutaneous fatty atrophy, depigmentation of the overlying skin, injection into an artery or vein instead of the joint, osteoporosis, acceleration of cartilage attrition, steroid flares (pain increased 72 hours after injection), steroid arthropathy, tendon rupture, facial flushing, iatrogenic infectious arthritis, and pericapsular calcification [7]. A prospective cohort study found that 50% of patients with subacromial bursitis responded to the local anesthetic injection presented with shoulder pain [8]. Though this prospective cohort study concluded there is no correlation between ultrasound findings and whether the injection worked, the fact that some patients with gene mutations and opioid users could be resistant to local anesthetics was not considered [8-9]. The causes for not responding to a particular treatment include gene mutations and opioid use. These factors were not analyzed in this study. Therefore, we cannot conclude whether the local anesthetics did not work or whether the individuals had certain factors that increased their local anesthetic resistance. 

Subacromial bursitis associated with fibromyalgia leads to more intense shoulder pain. The subacromial bursa has both nerves and pain mediators that enhance the pain. The subacromial bursa is innervated by the free nerve endings of suprascapular and lateral pectoral nerves in the subintima that lie close to the blood vessels [2]. Substance P release gives rise to pain in the subacromial bursitis. In the inflamed subacromial bursa of bursitis, the expression of vascular endothelial growth factor (VEGF) is the generator of pain during motion [2]. Tumor necrosis factor-alpha (TNF-α) is key in pain transmission associated with subacromial bursitis [10]. The administration of a transcapular nerve block into the bursa relaxes the myofascial trigger points in the supraspinatus and infraspinatus muscles, reducing the peripheral stimulus, which in turn can reduce substance P and the central sensitization of fibromyalgia [11].

Local anesthetic injection into the bursa can reduce the inflammation of bursitis by the following mechanism. As shown in Figure 1, inflammation activates the high mobility group box protein (HMGB1) both in the nucleus and the extracellular matrix. The nucleus all-thiol HMGB1 form upregulates the caspase-1 (inflammasome) and cytokine releases like interleukin (IL)-1β and IL-18. In the extracellular environment, di-sulfide HMGB1 is released actively from necrotic cells and passively from immune cells like macrophages, upregulating the toll-like receptor (TLR)-4 on the damaged cells of the bursa. The TLRs are pattern recognition receptor (PRR) sensors like security cameras watching for invaders. TLRs detect endogenous ligands (DAMPs) on the damaged cells of inflammation. This causes TLRs to activate by teaming up (dimerization) and sending messages via myeloid differentiation primary response protein 88 (MyD88) that activate protein kinases like p38 MAPK and IκB Kinase (IKK), a helper enzyme that switches on NF-κβ immediately (canonical pathway). NF-κB enters the nucleus and turns on genes that produce inflammatory signals, including pro-inflammatory cytokines (e.g., tumor necrosis factor (TNF)-α, IL-1) that attract immune cells fast to repair tissue; adhesion molecules (e.g., E-selectin, ICAM-1, VCAM-1) that help immune cells stick to blood vessel walls; chemokines (e.g., IL-8, MCP-1) that attract immune cells to the site of inflammation. The advanced glycation end products (RAGE) receptor-mediated pro-inflammatory signaling is also activated by extracellular HMGB1. In addition, TNF-α enhances ICAM-1 expression, aiding leukocyte adhesion and inflammation [12]. Local anesthetics (LA) reduce inflammation by modulating the activity of high mobility group box protein (HMGB1) both in the nucleus and the extracellular environment. In the extracellular environment, LA shifts di-sulphide HMGB1 towards a fully oxidized form and stabilizes the cell membrane, inhibiting HMGB1 release. Inhibited HMGB1 also diminishes receptors for advanced glycation end products (RAGE) mediated pro-inflammatory signaling [12]. LA targets signaling pathways like NF-κB, reducing the transcription of pro-inflammatory cytokines. It also interacts with p38 of MAPK, reducing pro-inflammatory cytokines. LAs inhibit ICAM-1 phosphorylation and tyrosine-protein kinase (Src) activation, further minimizing the TNF-α effect and associated pain [12]. A study suggests that LAs can effectively inhibit TNF-α production in human blood samples, demonstrating their anti-inflammatory potential in real-world clinical scenarios. This positions LAs as candidates for managing pain and inflammation in chronic inflammatory conditions [13].

The key takeaway from this case report is that the combination of transcapular nerve block with subacromial injection can provide better pain relief than other types of treatment alone. The transcapular nerve block targets the nerve supplying the shoulder. The subacromial bursa injection targets the inflammation in the bursa. This takes care of both sources of pain, improving function, alongside other fibromyalgia management strategies like exercises, medications, and stress management. In contrast, physical therapy can improve overall mobility and strength, and pain medications lower pain intensity. However, it may not be sufficient for significant shoulder pain.

This case report's strengths include laying the foundation for combining injections. It is easy to do, and no financial support is needed. A case report is observational and educational; however, it is limited by the study's design, which lacks generalization due to the lack of sample size and a control group for comparison. There can be selection bias in a case report.

## Conclusions

This case report investigated the use of a local anesthetic administered via a subacromial bursa injection along with a transcapular nerve block for shoulder pain management due to fibromyalgia and associated subacromial bursitis in a 53-year-old female patient. The report showed reduced VAS scores and S-LANNS scores. This case suggests that combined injections may be beneficial in select cases, but further studies are needed to confirm efficacy. The patient was also taking Gabapentin and Advil during the nerve blocks and subacromial bursa injections. The concomitant treatments could have a potential confounding effect. The diagnosis of subacromial bursitis was clinical in this case report. Diagnosing fibromyalgia-associated bursitis remains challenging, and some cases may require more objective diagnostic criteria, including more specific imaging like an MRI. The local anesthetic injected directly into the subacromial bursa in conjunction with the transcapular nerve block can potentially reduce shoulder pain commonly found in fibromyalgia by reducing inflammation and promoting tissue regeneration. Future large randomized controlled trials are needed to establish standard protocols and identify populations most likely to benefit from these injections. This case report was published with the consent of the patient.
